# Wild Boars (*Sus scrofa*, L. 1758) from Castile and Leon Region (Spain): A Histopathology Survey

**DOI:** 10.3390/ani12233282

**Published:** 2022-11-25

**Authors:** Catarina Jota Baptista, José M. Gonzalo-Orden, Luís J. Merino-Goyenechea, Paula A. Oliveira, Fernanda Seixas

**Affiliations:** 1 Department of Veterinary Sciences, School of Agrarian and Veterinary Sciences (ECAV), University of Trás-os-Montes and Alto Douro (UTAD), Quinta de Prados, 5001-801 Vila Real, Portugal; 2 Centre for Research and Technology of Agro-Environmental and Biological Sciences (CITAB), Inov4Agro, University of Trás-os-Montes and Alto Douro (UTAD), Quinta de Prados, 5000-801 Vila Real, Portugal; 3 Institute of Biomedicine (IBIOMED), University of León, 24071 León, Spain; 4 Departament of Biomedical Sciences, University of León, 24071 León, Spain; 5 Veterinary and Animal Research Center (CECAV), AL4Animals, University of Trás-os-Montes and Alto Douro (UTAD), Quinta de Prados, 5000-801 Vila Real, Portugal

**Keywords:** parasitic pneumonia, hydropic change, vacuolar change, histopathology

## Abstract

**Simple Summary:**

Eurasian wild boars (*Sus scrofa*) are species of interest to continuously study and monitor diseases due to their abundance, consumption, and their role as zoonotic disease reservoirs. To the authors’ knowledge, there is a lack of health assessments in this particular area of Spain, which represented an opportunity for this assessment. This study aims to report and interpret some histopathological findings (from the lung, liver, and kidney) in wild boars from different areas of Castile and León (Spain) to evaluate the health status of this population. Parasitic pneumonia (34.7%) in the lungs and cellular alterations (33.3%) in the liver are some of the most common and relevant lesions found. Further research and other diagnostic tests are needed to have definitive diagnoses or to estimate zoonotic disease prevalence.

**Abstract:**

Wild boars are wild ungulates with a wide distribution in Europe, with a relevant role in wildlife and public health. In Spain, high (and sometimes artificial) densities of wild boars are responsible for several health problems. Regular surveys, with hunters’ collaboration, are crucial to monitor these diseases. Histological analyses were performed for lung, liver, and kidneys from 72 wild boars (58 from Zamora, 16 from Palencia). Lungs were the most affected organs, mainly revealing parasitic pneumonia (34.7%). Hydropic, vacuolar, and other cellular changes (33.3%) and congestion (16.7%) were found in the liver, and only 30.6% of the wild boars presented no alterations in this organ. Regarding the kidney, non-purulent nephritis (22.2%) was the most common lesion. This study gives an overview of the health status of wild boar populations in Castile and León. Other laboratory analyses are needed to obtain definitive diagnoses of these lesions, reach other conclusions, or apply any mitigation strategies to protect animals’ or consumers’ health.

## 1. Introduction

*Sus scrofa* is a Eurasian wild ungulate with broad distribution in the Iberian Peninsula. Wild boars are recognized for their extraordinary adaptability to different habitats and high reproductive capacity. In addition, the absence of natural predators (such as wolves) or other significant threats, depopulation of the rural territories, and the abandonment of traditional agriculture contribute to boars’ occupation of new geographic areas. Consequently, they are currently overabundant in large areas of the Iberian Peninsula, and getting closer to humans, even in urban areas [1].

Most zoonotic diseases (71.8%) originate in wildlife [2]. Wild boars can be considered one of the best sentinel species in Mediterranean habitats. They are considered suitable surveillance targets in these areas, not only due to their abundance but also due to their accessibility for sampling, as a game species [3]. Therefore, wild boars’ studies, including pathology surveys, may provide relevant data about wildlife health in our ecosystems and public health, especially for hunters and boar meat consumers. The role of wild boars as sources of infection to humans has been discussed and published worldwide, regarding some zoonotic diseases, such as tuberculosis [4,5]. One of the most important settings of animal tuberculosis in Europe concentrates in the southwestern Iberian Peninsula, in which red deer, roe deer, and wild boar play an important role for its maintenance [6]. In Europe, the prevalence of tuberculosis in wild boar ranges from 1 to 52%, being higher in the southern Iberian Peninsula due to artificially high densities of wild boar (more than 90 per square km) and consequent group aggregation near feeders and waterholes [7]. However, other European wild boar diseases are also a matter of concern, as hepatitis E, presenting a prevalence of 9.5% in an Italian region [8], and 40.8% in western Bulgaria [9]. The importance of wild boars as sentinel species goes far beyond zoonotic disease surveillance, since they may also be considered suitable indicators of pollutant exposure [10].

Castile and León is the largest autonomous region of Spain, formed by a total of nine provinces (Ávila, Burgos, León, Palencia, Salamanca, Segovia, Soria, Valladolid, and Zamora) [11]. Animal tuberculosis is present and has been regularly assessed in wildlife in this region. However, wild boars’ prevalence in this region is considered low (4%), especially compared to other southwestern areas of Spain [6,12]. On the other hand, a human trichinellosis outbreak due to the consumption of wild boar meat from Castile and León was already reported [13]. A study performed in Castile and León (and Extremadura) reported a 65.2% global prevalence of *Metastrongylus* spp., a lung parasite of wild boars, as well as other relevant swine parasites as *Ascaris suum* or *Trichuris suis* [14]. Other diseases and infectious agents reported in some autonomous regions of Spain (including Castile and León) interfere with livestock disease control or affect human health, such as Aujeszky’s disease, porcine circovirus, *Brucella* spp., and *Toxoplasma gondii* [15].

Wildlife histopathology studies have been significantly contributing to our knowledge about animal and human diseases, providing a reference for reactive lesions to a certain agent or establishing a guide for comparative medicine [16,17], as well as being crucial to determine a cause of death or disease in a wild population [18].

To the authors’ knowledge, not many histopathology surveys with wild boars have been performed in this region. This work intends to show some lesions observed on the histopathology of the lung, liver, and kidney from hunted wild boars from the Castile and León region, Spain.

## 2. Materials and Methods

### 2.1. Sampling

A total of 72 wild boars culled between 7 and 25 February 2021 during the hunting season in Castile and León region, Spain, were included in the study: 58 wild boars were from different areas of Zamora province and 14 were from Palencia province (Appendix A; Table A1). No particular criteria, rather than population control and management, were applied to the animal selection. Approximately 20 g of liver, kidney, and lung were gently provided by hunters to ensure that we had enough amount of samples to perform our analysis. All the provided pieces were obtained from the central regions of the parenchyma of each organ (lungs and liver) and comprised all their layers. All the samples were collected from fresh carcasses just after the animal’s death, avoiding autolytic post-mortem changes that usually compromise the correct histopathological analysis. All the samples were fixed in 10% formalin and stored until transportation to the laboratory. Samples were analyzed in the Histopathology Laboratory from the University of Trás-os-Montes and Alto Douro (UTAD, Portugal), and processed for light microscopy by routine histologic technique. Samples were cut in small slices (2–3 mm) into tissue processing cassettes a in the fume hood (GrossLab, Shandon^®^, Thermo, Waltham, MA, USA). Tissues were then dehydrated and embedded in paraffin in a tissue processor (Dakewe HP300^®^, Shenzhen, China), and paraffin blocks were made in an embedding station (Histocentre, Shandon^®^, Thermo, Waltham, MA, USA). Histological slides were prepared in an automated microtome (Leica RM2255^®^, Leica Microsystems, Wetzlar, Germany) and stained with hematoxylin and eosin in a linear automated slide stainer with carousel (Varistain 24-4, Shandon^®^, Thermo, Waltham, MA, USA). Slides were observed under a blind test using an optical microscope (Nikon E600^®^, Nikon Instruments Inc., Melville, NY, USA).

### 2.2. Statistical Analysis

Microsoft Excel^®^ (accessed on 16 October 2022) was used to perform descriptive statistics. SPSS^®^ Statistics version 27.0 (accessed on 16 October 2022) was used for statistical inference analysis. Pearson Chi-square test and Fisher’s Exact test were used for qualitative data analysis. A confidence interval of 95% was considered (*p*-critical value = 0.05).

## 3. Results

### 3.1. Lung

**Figure 1:**
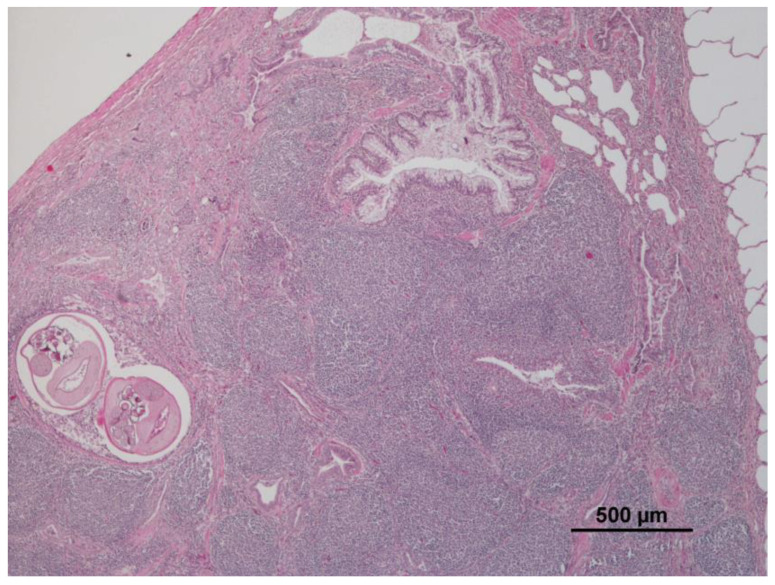
Severe lymphoid hyperplasia and verminous bronchitis (10×, 4×).

A lot of animals presented verminous bronchitis (25/72; 34.7%) and showed bronchial and bronchiolar lumens filled with nematodes associated with catarrhal inflammation, free mucous, and severe lymphoid hyperplasia of Bronchus-Associated Lymphoid Tissue (BALT) (Table 1). There was also severe to moderate hypertrophy and hyperplasia of the bronchus and bronchiolar smooth muscle and occasional thickening of alveolar walls. Eosinophils in the interstitial tissue, as well as in the bronchial and bronchiolar walls were also observed. More rarely, small granulomas with giant cells and eosinophils surrounding eggs or larvae, alveolar edema, and atelectasis (14/72; 19.4%) were seen (Figure 1). However, some animals presented hyperplasia of BALT and hypertrophy and hyperplasia of the bronchus and bronchiolar smooth muscle without any observed nematodes.

Hemorrhage, with no associated organic response, was detected in 34.7% of the cases (25/72). Other lung lesions such as emphysema, alveolar proteinosis, and the presence of silicates and hemosiderin were also observed, although in lower proportion than others. Only 13 animals did not show lung changes (13/72; 18.1%). No significant differences were found between the presence of each lesion and a specific region or province.

### 3.2. Liver

**Figure 2:**
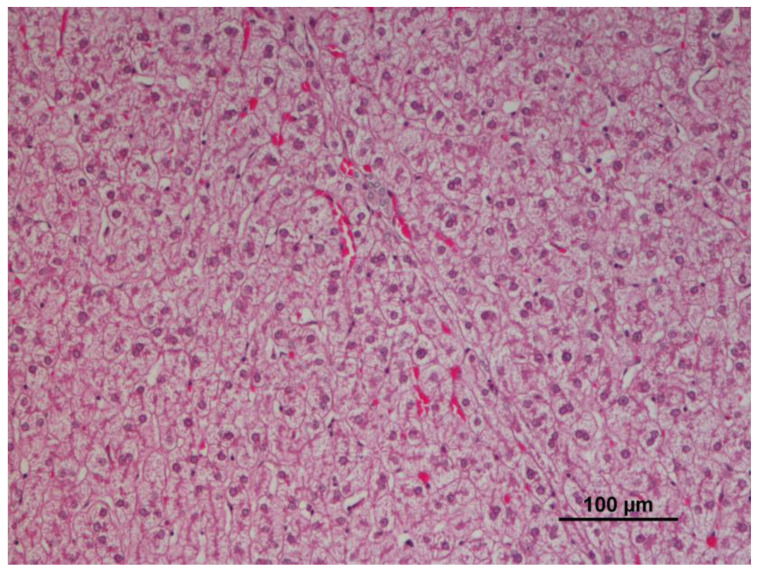
Panlobular hydropic change in the liver (10×, 10×).

Mild to moderate cellular changes, including cell swelling, hydropic change, or vacuolar change, were the most common findings in the liver of the wild boars (24/72; 33.3%) (Figure 2), due to intracellular glycogen storage. Congestion of the majority of the parenchymal blood vessel (12/72; 16.7%) and infiltrations of eosinophils (11/72; 15.3%) (Table 1) were also observed. An increase of other white blood cells, hemosiderin, granulomatous hepatitis, subacute hepatitis, and steatosis were also observed, although with prevalence of less than 7%.

In 22 animals, there were no significant liver changes (22/72; 30.6%). A statistically significant difference of the presence of cellular changes was found between Zamora and Palencia provinces (*p* = 0.003). In fact, all 24 cases were found in Zamora and no case was found in Palencia province.

### 3.3. Kidney

**Figure 3:**
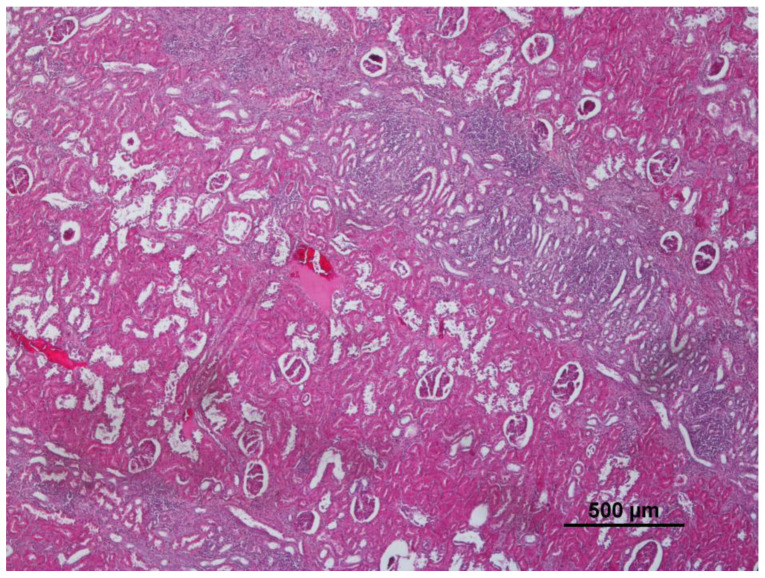
Interstitial nephritis detected in the kidney (10×, 4×).

Compared to the other organs, the kidney revealed fewer changes, and 34 wild boars presented no detectable alterations (34/72; 47.2%). Non-purulent (subacute) interstitial nephritis was the most frequently found lesion in the kidney (16/72; 22.2%), characterized by a generalized inflammation of renal interstitium, with the presence of inflammatory cells and fluid surrounding the renal tubule (Figure 3). Congestion of the majority of the parenchymal blood vessel was present in 11 wild boars (11/72; 15.3%) (Table 1). Renal steatosis, chronic interstitial nephritis, basal membrane thickening, hemosiderin, and purulent nephritis were also observed, though in two or fewer wild boars. No significant differences were found between the presence of each lesion and a specific region or province.

## 4. Discussion

In our study, the lung, kidney, and liver of 72 wild boars killed during the hunting season of February 2021, in Zamora and Palencia (Castile and León, Spain) were analyzed. Lungs were the most affected organs, and lesions were seen in more than 80% of the wild boars. Wild boars frequently migrate in groups of females or lone males. Nevertheless, this territorial expansion may contribute to an intraspecific (especially between females) and interspecific (with domestic or wild species) spread of infectious diseases and, consequently, histopathology lesions may be found [19].

Histologically, verminous pneumonia was the most prevalent pathology observed. It was mainly associated with hyperplasia of BALT, bronchus and bronchiolar smooth muscle hyperplasia, and with the presence of nematodes inside bronchus and bronchioles. Lymphoid hyperplasia of the lung belongs to the spectrum of reactive pulmonary lesions [20], usually associated with parasitic lesions. *Metastrongylus* spp. infections in wild boars is associated with the thickening of alveolar walls, infiltration of the interstitial tissue, and lymphoid hyperplasia [21]. In some of our cases, infected lungs presented hyperplasia of BALT, as well as hypertrophy and hyperplasia of the smooth muscle, but no nematodes were observed. This may be due to sampling of non-representative areas of the lung, or due to parasite infection recovery before death. A study performed in Castile and León (and Extremadura) reported 65.2% global prevalence of *Metastrongylus* spp.). Parasitological exams are necessary to identify the nematodes present in our lung samples.

Enzootic pneumonia (*Mycoplasma hyopneumoniae*) has also been mentioned as a common cause of BALT hyperplasia in wild boars, which presents reactive lymphoid follicles, increased bronchial and alveolar neutrophils, and interstitial infiltrates of different cells (plasma cells, histiocytes and lymphocytes) [22]. A recent study also reported these histopathological changes as the most prevalent in wild boars from southern Brazil. BALT hyperplasia was present in 86.5% of the 226 cases, followed by suppurative bronchopneumonia (65.7%). Bacterial agents were also tested, and BALT hyperplasia was statistically associated with *M. hyopneumoniae* and *M. hyorhinis* [23].

The observed lung hemorrhages (34.7%) were perimortem (agonic), not associated with acute inflammatory response, so they may be associated with pulmonary contusions induced by high-velocity gunshot injuries [24]. Nevertheless, important swine infectious diseases (as African swine fever) are also associated with multiple hemorrhages, including in the lungs [25], but other organic lesions are usually present. Hemosiderin may be associated with old hemorrhagic lesion. Thus, hemorrhagic lesions must also be considered in hunted wild boars’ sanitary inspection, and other causes (rather than gunshot injury) must not be immediately discarded.

Hydropic and vacuolar changes were the most common liver changes observed and were associated to glycogen storage, suggesting good nutrition of these wild boars [26]. However, stress, bacteria, virus, and toxic chemical substances (as persistent organic pollutants, mercury, cadmium, and lead) are among other possible causes of hydropic changes in multiple organs, including the liver [22,27]. Due to the lack of specificity of these alterations, it is complicated to explain the statistical difference found between Zamora and Palencia. Variations in food availability between regions may be responsible for differences in hepatic glycogen and consequent hydropic changes. On the other hand, the frequent use and detection of chlortoluron (and other herbicides) in soils, especially in cereal production in Zamora, may be another explanation for these differences in hydropic hepatic change [28,29]. The use of herbicides and their (direct or indirect) consumption is a health hazard for both animals and humans. Nevertheless, only 14 wild boars were sampled in Palencia, compared to 58 in Zamora, which might interfere with this statistic result. Further research, mainly biochemical and toxicological exams, are needed to support any of these causes.

Higher amounts of eosinophils in the liver (15.3% of the cases) are probably associated with parasitic infections, although no parasites have been observed in our liver samples. *Ascaris suum*, *Fasciola* spp. (as *F. hepatica*), *Dicrocoelium dendriticum*, or *Echinococcus multilocularis* are some examples of parasites that may affect wild boars’ liver [30,31,32]. In addition to their importance in other domestic and wild animals, the mentioned parasites have been reported as having zoonotic potential, leading (more or less frequently) to human disease outbreaks [33,34,35]. Therefore, manipulating wild boar carcasses requires protective health measures (as the use of personal protective equipment) [36]. Other causes, such as allergic diseases or drug hypersensitivity, may also be related to high levels of eosinophils in the liver [37] and other organs. Congestion and increase of different white-blood cells that we also detect suggest inflammation of unspecified caused.

The kidney was the less affected organ (compared to the lungs and liver), presenting no detectable lesions in 47.2% of the wild boars. Non-purulent nephritis was the most frequent lesion (16; 22.2%). Porcine circovirus type-2 (PCV2) is responsible for severe non-purulent interstitial nephritis in wild boars and domestic pigs worldwide [38,39]. Another cause of kidney disease in wild boars is infection by the zoonotic bacteria *Leptospira* spp. Leptospirosis was detected in 18% of 141 urban wild boars in a study in Berlin, Germany, and was associated with chronic interstitial nephritis [40]. The presence of this bacteria in an urban environment reinforces the importance of health monitoring and preventive measures to avoid the infection of domestic species and humans [41]. In fact, the correlation between the presence of *Leptospira* spp. in wild populations and disease outbreaks in humans has already been proven [42,43]. Chronic nephritis was present in two wild boars in the present study (2.8%), therefore testing these animals for *Leptospira* spp. would be adequate to determine the possible cause of the lesions observed.

Hemosiderin was found in all three organs (lung, liver, and kidney), although in a few wild boars. Hemosiderin can accumulate in different organs in various diseases. Although it is an unspecific finding, it is especially abundant after a hemorrhage in the tissue, suggesting phagocytosis of red blood cells and hemoglobin [44].

Other diagnostic tests are needed to find the definitive cause of these histopathological lesions, as molecular analysis, bacterial cultures, or toxicological methods. An integrated analysis allows a deeper understanding of health threats affecting *S. scrofa*, as well as its role in disease spreading to other wildlife, domestic species, and humans. Although with a limited capacity to draw definitive conclusions, even the simplest (but well-performed) wildlife pathology survey provides an idea about the general population’s health status [17]. There are not many histopathological studies of the wild boar populations in this region. It is relevant to start with a general health approach (namely with histopathology) to follow the next step to a more specific disease monitoring plan. Therefore, this study may provide a first orientation to what should be prioritized, or what surveys should be carried out in these wild boars.

## 5. Conclusions

For all three organs (lung, liver, and kidney), the number of samples with histological alterations is higher than those with a normal pattern. Overall, the lung was the organ where more lesions were identified, followed by the liver. Pulmonary infection by lungworms associated with lymphoid and smooth muscle hyperplasia was the most common lesion. Cellular changes (hydropic and vacuolar changes) in hepatocytes were found in a third of the cases, mostly associated with glycogen. Non-purulent nephritis was found in the kidneys of 16 wild boars.

Some histopathological lesions found are compatible with the possible presence of some zoonotic agents with public health relevance (namely *Metastrongylus* app., *Leptospira* spp., or liver fluke worms). This emphasizes the importance of adopting protective measures by hunters, veterinarians, or consumers when contacting wild boar fresh carcasses.

Other laboratory analyses are necessary to find the etiology of these lesions and evaluate the potential public health threat (if, for instance, zoonotic pathogens are involved). Nevertheless, histopathological reports and collaboration between veterinarians and hunters have considerable relevance and may work as an encouragement for more profound research and surveillance plans.

## Figures and Tables

**Table 1:** Histopathologic lesions found in each organ (lung, liver, and kidney), as well as the number (N) and percentage (%) of animals affected.

Lung			Liver			Kidney		
Lesions	N	%	Lesions	N	%	Lesions	N	%
Lymphoid hyperplasia	26	36.1	Cellular changes: cell swelling; hydropic change; vacuolar change	24	33.3	Non-purulent nephritis	16	22.2
Agonic hemorrhage	25	34.7	centrolobular	1	1.4	Congestion	11	15.3
Verminous bronchitis	25	34.7	panlobular	8	11.1	Renal steatosis	2	2.8
Atelectasis	14	19.4	diffuse/not specified	15	20.8	Chronic interstitial nephritis	2	2.8
Congestion	6	8.3	Congestion	12	16.7	Basal membrane thickening	2	2.8
Emphysema	4	5.6	Increase of eosinophils	11	15.3	Hypercellular glomeruli	2	2.8
Alveolar proteinosis	1	1.4	Increase of other white cells	5	6.9	Cellular infiltration	2	2.8
Silicates	1	1.4	Hemosiderin	4	5.6	Hemosiderin	2	2.8
Hemosiderin	1	1.4	Granulomatous hepatitis	1	1.4	Purulent nephritis	1	1.4
			Subacute hepatitis	1	1.4			
			Steatosis	1	1.4			

## Data Availability

Not applicable.

## Appendix A. Location of the Sampled Wild Boars

**Table A1:** Location and number of wild boar samples (N) from each location.

Province	Location	N
Zamora	Granucillo	12
	Friera de Valverde	10
	Ferreruela	9
	Melgar de Tera	7
	Perilla de Castro	6
	Vegalatrave	3
	Argañin	2
	Montamarta	2
	Dehesa Pozos/Tabara	1
Palencia	San Cebrian de Campos	14
